# Cognitive-motor integration deficits in young adult athletes following concussion

**DOI:** 10.1186/s13102-015-0019-4

**Published:** 2015-10-19

**Authors:** Jeffrey A. Brown, Marc Dalecki, Cindy Hughes, Alison K. Macpherson, Lauren E. Sergio

**Affiliations:** 1 School of Kinesiology and Health Science, York University, 357 Bethune College, 4700 Keele Street, Toronto, M3J 1P3 ON Canada; 2 Centre for Vision Research, York University, Toronto, Canada; 3 York University Sport Medicine Team, York University, Toronto, Canada; 4 Southlake Regional Health Centre, Newmarket, ON Canada

**Keywords:** Mild traumatic brain injury, Prediction model, Movement control, Return to play protocol, Eye-hand coordination

## Abstract

**Background:**

The ability to perform visually-guided motor tasks requires the transformation of visual information into programmed motor outputs. When the guiding visual information does not align spatially with the motor output, the brain processes rules to integrate the information for an appropriate motor response. Here, we look at how performance on such tasks is affected in young adult athletes with concussion history.

**Methods:**

Participants displaced a cursor from a central to peripheral targets on a vertical display by sliding their finger along a touch sensitive screen in one of two spatial planes. The addition of a memory component, along with variations in cursor feedback increased task complexity across conditions.

**Results:**

Significant main effects between participants with concussion history and healthy controls without concussion history were observed in timing and accuracy measures. Importantly, the deficits were distinctly more pronounced for participants with concussion history compared to healthy controls, especially when the brain had to control movements having two levels of decoupling between vision and action. A discriminant analysis correctly classified athletes with a history of concussion based on task performance with an accuracy of 94 %, despite the majority of these athletes being rated asymptomatic by current standards.

**Conclusions:**

These findings correspond to our previous work with adults at risk of developing dementia, and support the use of cognitive motor integration as an enhanced assessment tool for those who may have mild brain dysfunction. Such a task may provide a more sensitive metric of performance relevant to daily function than what is currently in use, to assist in return to play/work/learn decisions.

## Background

Concussion can be defined as a rapid onset brain injury leading to short-lived impairment of neurological function that resolves spontaneously [1]. With concussion, function may be interrupted but there is no obvious structural damage to the brain using current structural neuroimaging techniques [2], although recent imaging and behavioral studies have found alterations in both function and anatomy in particular brain regions following concussion [3–5]. Moreover, concerns over the short and long term effects of the concussion suffered by professional athletes have been highly influential in bringing concerns about concussion in sport to the global scale. Recent media attention has focused on the short-term memory loss, headache, and migraine suffered 10–20 years following concussion in sports such as football and hockey [6]. Recent behavioral studies have found up to a five-fold increase in mild cognitive impairment (MCI) and earlier onset of Alzheimer’s disease (AD) among retired football athletes [7]. It is also recognized that concussions are cumulative; once one has had a concussion, it is easier to get another one, with symptoms often lasting longer. For example, football players with three concussions were three times as likely to suffer another one relative to a player with no concussion [8, 9]. The neurological reasons underlying this increased vulnerability are poorly understood.

While at present the Return to Play or Return to Work protocols are fairly well-established, there remains a lack of robust metrics for use by clinicians to thoroughly assess function following a mild brain injury prior to safe resumption of pre-injury activities [1]. Current neurocognitive testing reports (balance, vestibular, oculomotor, symptoms, neuropsychological tests) measure cognitive and motor abilities separately. However, in daily life, and particularly during many sport activities, there occur several situations where the brain needs to integrate both cognition and movement control concurrently. This rule-based motor performance can occur for example when the brain has to decouple vision from action (e.g., gaze and hand motion are in different directions and spatial locations) [10]. One might argue that if the brain is ‘pushed’ to think and act concurrently following a concussion, there may occur functionally relevant performance deficits, ones which currently used metrics testing cognitive and motor abilities separately do not find. If so, more sensitive performance metrics testing cognitive and motor abilities concurrently could, arguably, reduce the potential for re-injury.

Previously, our group has indeed examined rule-based motor performance (“cognitive-motor integration”) in adults affected by age-related mild brain dysfunction [11–14]. Such integration is often required when performing non-standard visuomotor tasks, where a rule is used to align the required motor output to the guiding visual information [15]. A standard task refers to one which involves direct interaction with an object, such as reaching for a coffee cup in front of you; the eye and hand end at the same spatial location. A non-standard task results in different final spatial locations for the eye and hand, such as making a horizontal movement with a computer mouse in order to vertically displace the cursor viewed on the monitor. In our research to date we have observed that reaching and gross motor movements made under congruent, standard conditions are *not* impaired in early Alzheimer’s disease (eAD) and mild cognitive impairment (MCI) relative to healthy aging. Significantly, however, we have found that as soon as an element of dissociation is introduced into a reaching task (the guiding visual information is spatially decoupled from the required motor act, such as using a computer mouse or parking a car using a rear-view mirror), eAD performance declines precipitously relative to healthy adults, whose performance also declines but much less so [11, 16]. Our more recent results suggest that adults with MCI, and even healthy adults with a familial dementia risk, also show a decline in performance, albeit less dramatically [12–14]. Thus there appears to be an impaired ability to integrate rules into coordinated motor tasks with the presence of mild brain dysfunction.

To date, there has been little research examining the utility of assessing mild brain dysfunction brought on by concussion using cognitive-motor integration [17]. Concussion’s longer term effects on cognitive ability and cognitive-motor integration are poorly understood and not fully characterized. Indeed, there is a limited amount of information on functional problems associated with having a history of concussion. A more sensitive quantification of function post-concussion would in turn assist in return-to-play/work/learn decisions. In the present study, we address this gap in knowledge by studying the performance of university varsity athletes both with a history of concussion and healthy age matched controls without concussion history on a movement coordination task requiring rule integration. Based on our previous work, we hypothesize that cognitive-motor integration is affected in athletes with a history concussion when compared to healthy age-matched controls without concussion history. Specifically we predict that, like older adults at risk for developing dementia, these athletes with concussion history will show degraded movement planning and movement execution performance when there are two or more levels of decoupling between vision and action. To find out, we used in the present study the same cognitive-motor integration task as in our previous work, which has proven to produce valid and reliable data and to be effective at quantifying subtle cognitive-motor integration changes in those at risk of, or in the early stages of dementia [11–13, 16]. Therefore, it was an additional aim of our study to assess the effectiveness of our computer-based task and to find out if we are able to distinguish between participants with concussion history and with no-history of concussion. Here we report that in support of our prediction, athletes with a concussion history - most of whom were asymptomatic by current measures-nevertheless displayed significant performance impairment when required to think and move at the same time.

## Methods

### Participants

We recruited 18 athletes with a history of concussion (Age: 21.44 ± 4.29 years; 2 female, 16 male) and 17 healthy control participants (Age: 20.44 ± 2.43 years; 9 female, 8 male) for this study. All participants of both groups were recruited from the York Lions varsity sport teams (including football, hockey, rugby, basketball, volleyball, track and field and field hockey) at York University Toronto, ON, Canada. Potential participants were approached during their routine pre-season baseline medical testing, and their concussion history was unknown to the experimenter at the time of testing. The participants were free of neurological conditions excluding concussion history. All participants’ concussions were being managed by the York University Sport Medicine team. Within the concussion history participant group, 13 were asymptomatic and were already progressing through the return to play protocols at the time of testing, and five participants were still symptomatic at the time of testing and had not begun the Return to Play protocols. Details for the participants with a history of concussion are summarized in Table 1. All participants signed a consent form to participate in the experiment, approved by the York University review board.

**Table 1:** Characteristics and concussion incidence for participants with concussion history

Part.	Age	# of Conc.	Time since last Conc. (months)	Symp./Asymp.	Symptoms	Sport
1	21	1	2	Asymp.	-	B
2	19	1	7	Asymp.	-	H
3	34	8	5	Symp.	Headache, light-sensitivity, fatigue	H
4	20	3	0.5	Asymp.	-	H
5	22	6	15	Asymp.	-	F
6	19	2	0.5	Asymp.	-	H
7	18	1	84	Asymp.	-	F
8	20	1	0.75	Symp.	Headache, dizziness	V
9	27	1	0.25	Symp.	Headache, light-sensitivity, dizziness	NV
10	22	1	6	Asymp.	-	F
11	18	2	9	Asymp.	-	F
12	23	3	48	Asymp.	-	F
13	20	2	48	Asymp.	-	F
14	18	1	108	Asymp.	-	F
15	21	1	0.25	Asymp.	-	FH
16	18	1	0.5	Symp.	Headache	R
17	28	1	0.1	Symp.	Headache	NV
18	18	4	1	Asymp.	-	F
Mean ± SD	21.4 ± 4.3	2.2 ± 1.9	18.66 ± 32.07			

*Part*. participant, # number, *Conc*. concussion, *Symp*. symptomatic, *Asympt* asymptomatic, *B* basketball, *H* hockey, *F* football, *V* volleyball, *NV* non-varsity, *FH* field hockey, *R* rugby

### Procedure

While performing the experiment, participants were seated at a desk so that they could comfortably reach a laptop computer with two touch-sensitive screens, one in the vertical and one in the horizontal plane (ACER brand computer, Model: Iconia 6120). For all conditions in the experimental task, participants were instructed to slide their index finger along one of the touch screens in order to displace a cursor as quickly and accurately as possible from a central circle to one of four peripheral targets (all 20 mm diameter) that were presented on the vertical screen. The targets were located 75 mm directly to the left, right, above, or below the home target. A yellow home target was presented in the center of the vertical tablet and participants touched the home target (either directly or with the cursor using the horizontal tablet depending on the condition), which then changed to green. After holding the home target for 4000 ms a red peripheral target was presented and the home target disappeared, serving as a ‘go-signal’ for participants to look towards the visual target and slide their finger along the touchscreen in order to direct the cursor to the target. Once the cursor reached the peripheral target and remained for 500 ms, it disappeared and the trial ended. The next trial began with the presentation of the home target after an inter trial interval of 2000 ms. To ensure smooth movement of the finger during the experiment task, participants wore a capacitive-touch glove on their preferred hand.

**Fig. 1:**
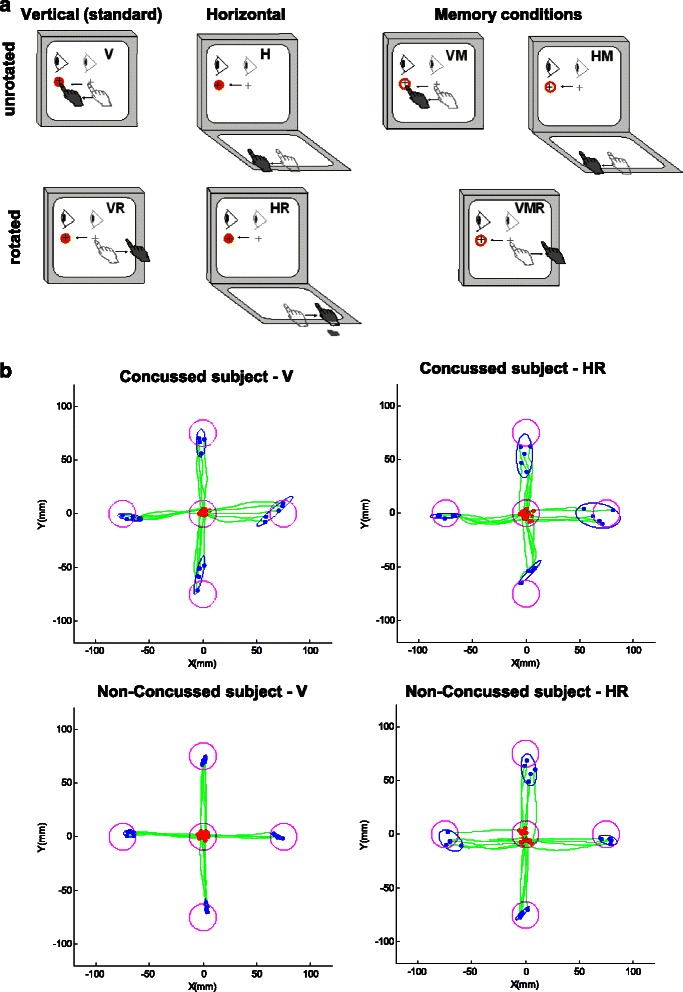
Drawing of experimental conditions and example of typical hand path data. **a** Schematic drawing of the experimental conditions. Visual stimuli were presented on the vertical monitor for all conditions. Light grey cursor, eye, and hand symbols denote the starting position for each trial (home target). Dark grey eye and hand symbols denote the instructed eye and hand movements for each task. Red circles denote the peripheral (reach) target, presented randomly in one of four locations (left, up, right, down). The dark crosshair denotes the cursor feedback provided during each condition. The open circles denote the cued position before the movement (seen in memory conditions). **b** Typical hand path data of one participant with concussion history and one participant with no-history of concussion performing the V and HR condition. Note the poorer performance in condition HR compared to V, particularly in the concussed participant

Different experimental conditions with different levels of decoupling of vision and action were used. The decoupling was achieved by either changing the plane on which the finger moved (vertical V, or horizontal H), changing the direction of cursor visual feedback (veridical or 180° rotated R, i.e. to move cursor right, slide finger left.), or inserting a memory delay (M) between target presentation and go signal (cf. Fig. 1a). These conditions were performed in a randomized block design. All combinations of the spatial correspondence, visual feedback, and memory conditions were tested to make seven conditions: V (vertical), VR (vertical rotated), VM (vertical memory delay), VRM (vertical rotated memory delay), H (horizontal), HR (horizontal rotated), and HM (horizontal memory delay). There were a total of 140 trials for each participant (4 directions × 5 trials × 7 conditions). Note that within the seven conditions only V represents a standard visuomotor mapping, where eye and hand movements were congruent in direction and plane. All other conditions required cognitive-motor integration in order to successfully perform a movement where the effector was decoupled from the guiding visual information. In the memory conditions, the target center appeared, and once the participants positioned the cursor in the central target, the peripheral target stayed for 2000 ms, followed by the removal of the peripheral target. The participants had to hold the cursor within the target for an additional 2000 ms and then it disappeared, serving as a go signal to move to the remembered target location.

Prior to each experimental condition, participants were allowed two practice trials in each direction (i.e., eight practice trials for each condition). Due to the possibility of cervical soft-tissue injury following concussion and the need for portability, eye-tracking was not feasible. Participant’s eye movements were monitored by the experimenter and if incorrect movements were made in the non-standard conditions, participants were reminded to always look towards the target and not at their hand; these trials were excluded from further data analyzing.

### Data processing and analysis

#### Movement trajectories and timing

The custom-written (C++) acquisition software used the touch-screen computer’s internal CPU clock to align the finger’s X-Y screen position to exact sampling times. The sampling rate was approximately 55 Hz. Individual movements paths derived from the cursor location were first low-pass Butterworth filtered at 10Hz (filfilt function, Matlab, Mathworks Inc.). Custom software was then used to generate a computerized velocity profile of each trial’s movement, with movement onset and end being recorded at 10 % peak velocity. These profiles were then verified by visual inspection and corrections were performed when necessary. Reaction time (RT) was calculated as the time interval (milliseconds; ms) between the central target disappearance and the point at which the finger velocity reached 10 % peak velocity. Movement time (MT) was the time (milliseconds; ms) between onset and offset, thereby representing the ‘ballistic’ initial movement without corrective adjustments. Pathlength (PL) was recorded for each trial, and was quantified as the distance (millimeter; mm) between start and the first correction of the initial cursor movement. The constant error (CE, i.e., movement accuracy) was determined as the distance (millimeter; mm) between the average movement endpoint for each target location (∑ x/n, ∑ y/n) and the actual target central location. Variable error (VE, i.e., movement precision), was determined as the distance (millimeter; mm) between the endpoints of the individual movements (σ2) from their mean movement endpoint.

#### Direction reversals and task completion error counts

Direction reversals errors (DR) were calculated when there was a deviation of more than ±45° from the line between center of central and peripheral target during the first half of each movement. Failed trials were counted for each condition. Failures were: failure to start trial within 10,000 ms of onset, failure to remain in the central target for 2000 ms (4000 ms M conditions), leaving start < 150 ms after the go signal, leaving the start > 5,000 ms after the go signal, or exceeding the maximum movement time to a target (>10,000 ms).

### Statistical analyses

For all dependent variables, main effects of Group (Non-history, Concussion history) and Condition (V, VR, VM, VRM, H, HR, HM) were analyzed using repeated-measures mixed ANOVA. Additionally, initial separate analyses using sex or symptomatic/asymptomatic as a main effect were run. Trials whose value was >2 standard deviations (SDs) away from the mean for a given condition in a given group were considered outliers and removed before statistical analysis. All remaining data were checked for normal distribution (Shapiro-Wilk’s test) and sphericity (Mauchly’s test), and were Greenhouse-Geisser corrected where necessary. Statistical significance levels were set to 0.05, and all analyses were performed using SPSS software (IBM corp.).

### Level of dissociation

We grouped the conditions by ‘level of eye-hand dissociation’ in order to test our hypothesis that performance declines would be significant for only the most decoupled situations. For this analysis, we compared participants’ performance as a function of their change from the most direct condition (no decoupling, standard mapping), similar to our previous studies. Specifically, we subtracted out the result on the V condition from the other six conditions for a given dependent measure. We grouped these six ‘delta’ conditions into 1 level (VM, H, VR) and 2 levels (HM, VRM, HR) of dissociation and performed a repeated-measures mixed ANOVA with the within factor Level (1 level, 2 levels) and the between factor Group (No-history, Concussion history).

### Discriminant analysis

In order to test if our task was sensitive enough to predict a presence of concussion history based on performance, we performed separate stepwise discriminant analyses testing different combinations of dependent measures between the no-history of concussion and the concussion history group.

## Results

The two analyses using sex or symptomatic/asymptomatic status as a main effect yielded no significant differences for any dependent variable (all p > 0.05), supporting the decision to merge data across sexes and status. Examples of typical movement trajectories of one subject with concussion history and one subject with no-history of concussion performing the V and HR conditions are presented in Fig. 1b-e. Statistical outcomes of the repeated mixed measures ANOVA for Group (No-history, Concussion history) and Condition (V, VR, VM, VRM, H, HR, HM) and significant pair-wise comparisons are summarized in Table 2, and the according descriptive statistics in Table 3.

**Table 2:** Statistical outcome repeated-measures mixed ANOVA of Group (Concussion history, No-history) and Condition (V, VR, VM, VRM, H, HR, HM) for all dependent variables (RT, MT, PL, CE, VE, DR)

Parameter	Group	Condition	Group × Condition
RT	F (1,33) = 9.28^**^	F (6, 198) = 20.77^***^	F (6, 198) = 1.56^n.s.^
MT	F (1,33) = 15.25^***^	F (6, 198) = 11.79^***^	F (6, 198) = 3.59^**^
PL	F (1,33) = 3.75 ^n.s.^	F (6, 198) = 2.94^**^	F (6, 198) = 0.53^n.s.^
CE	F (1,33) = 1.55 ^n.s.^	F (6, 198) = 10.30^***^	F (6, 198) = 0.84^n.s.^
VE	F (1,33) = 1.15 ^n.s.^	F (6, 198) = 12.30^***^	F (6, 198) = 3.30^**^
DR	F (1,33) = 1.80 ^n.s.^	F (6, 198) = 1.13 ^n.s.^	F (6, 198) = 0.64 ^n.s.^
Parameter	Condition	Group	
RT	VR	F (1,33) = 5.48^*^	
	VM	F (1,33) = 6.70^*^	
	VRM	F (1,33) = 7.51^*^	
	HM	F (1,33) = 13.50^**^	
MT	V	F (1,33) = 7.38^*^	
	VRM	F (1,33) = 19.47^***^	
	H	F (1,33) = 7.32^*^	
	HM	F (1,33) = 9.62^**^	
VE	V	F (1,33) = 10.53^**^	
	VR	F (1,33) = 8.94^**^	
	H	F (1,33) = 17.63^***^	
	HR	F (1,33) = 4.52^*^	

Significant outcomes of pair-wise comparisons for Group (Concussion history, No-history) of all variables and conditions

*RT* reaction time, *MT* movement time, *CE* endpoint accuracy, *VE* endpoint precision, *DR* direction reversal errors, *PL* pathlength, *V* vertical, *VR* vertical rotated, *VM* vertical memory, *VRM* vertical rotated memory, *H* horizontal, *HR* horizontal rotated, *HM* horizontal memory, *n.s*. non significant. Asterisks represent * < 0.05, ** < 0.01, *** < 0.001

**Table 3:** Descriptive statistics of main repeated-mixed ANOVA for all conditions vand groups (Concussion history, No-history) of all dependent variables (RT, MT, PL, CE, VE, DR)

Parameter	Condition	No-history	Concussion history
		[ms]	[ms]
RT	V	392.04 ± 90.98	426.82 ± 65.90
	VR	470.86 ± 69.63	548.87 ± 119.50
	VM	376.45 ± 105.34	466.05 ± 99.49
	VRM	369.49 ± 88.06	451.99 ± 89.04
	H	403.92 ± 87.56	443.80 ± 92.98
	HR	497.75 ± 98.40	536.55 ± 62.10
	HM	336.76 ± 74.29	440.56 ± 91.41
MT	V	336.53 ± 91.07	436.03 ± 122.31
	VR	475.99 ± 178.49	554.01 ± 160.76
	VM	466.45 ± 247.88	550.76 ± 174.42
	VRM	450.10 ± 163.19	775.07 ± 225.34
	H	402.15 ± 189.70	573.52 ± 184.97
	HR	526.44 ± 278.17	710.44 ± 270.83
	HM	427.62 ± 192.04	680.46 ± 279.42
		[mm]	[mm]
PL	V	88.88 ± 4.58	89.21 ± 2.91
	VR	88.57 ± 6.00	89.51 ± 6.27
	VM	89.88 ± 5.61	92.03 ± 5.03
	VRM	88.95 ± 8.98	90.18 ± 4.66
	H	84.40 ± 7.66	88.27 ± 6.82
	HR	87.60 ± 5.44	90.70 ± 5.53
	HM	86.89 ± 4.96	88.34 ± 5.05
CE	V	10.67 ± 2.93	9.51 ± 2.90
	VR	14.05 ± 3.14	14.88 ± 4.84
	VM	10.87 ± 3.17	10.73 ± 3.86
	VRM	12.70 ± 3.16	15.13 ± 5.27
	H	13.04 ± 4.53	15.51 ± 4.44
	HR	14.02 ± 3.55	15.58 ± 4.63
	HM	12.84 ± 4.09	13.67 ± 4.46
VE	V	6.90 ± 1.17	9.71 ± 3.38
	VR	6.53 ± 1.51	8.35 ± 2.04
	VM	9.41 ± 1.78	9.95 ± 2.93
	VRM	10.27 ± 1.91	10.07 ± 3.10
	H	6.77 ± 1.12	10.45 ± 3.44
	HR	8.01 ± 2.79	9.88 ± 2.40
	HM	8.18 ± 1.81	8.16 ± 2.30
		[count]	[count]
DR	V	0.00 ± 0.00	0.17 ± 0.38
	VR	0.18 ± 0.53	0.44 ± 1.25
	VM	0.12 ± 0.33	0.06 ± 0.24
	VRM	0.00 ± 0.00	0.28 ± 0.75
	H	0.06 ± 0.24	0.00 ± 0.00
	HR	0.12 ± 0.33	0.28 ± 0.96
	HM	0.06 ± 0.24	0.17 ± 0.51

*RT* reaction time (ms), *MT* movement time (ms), *CE* endpoint accuracy (% target distance), *VE* endpoint precision (% target distance), *DR* direction reversal errors (count), *PL* pathlength (mm), *V* vertical, *VR* vertical rotated, *VM* vertical memory, *VRM* vertical rotated memory, *H* horizontal, *HR* horizontal rotated, *HM* horizontal memory

### Performance timing

**Fig. 2:**
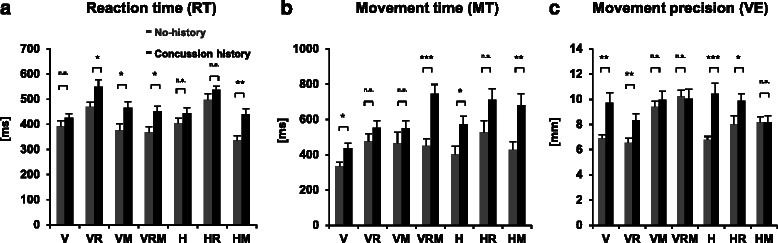
Mean movement timing and execution values for both groups (Concussion history, No-history) across all experimental conditions. Summarized are variables that showed a significant group effect, for movement timing **a** reaction time, and **b** movement time, and for **c** endpoint precision. Note the impaired movement timing and execution performance for participants with concussion history compared to participants with no-history of concussion. Abbreviations: V = vertical; M = memory; R = rotated feedback, H = horizontal, n.s. = non-significant. Asterisks represent *p < 0.05, **p < 0.01, ***p < 0.001. Error bars represent standard error of the mean

Repeated-measures mixed ANOVA revealed significant effects for RT for both Group (*p* < 0.01) and Condition (*p* < 0.001), but not for Group × Condition (*p* > 0.05). ANOVA yielded a main effect of Group (*p* < 0.001) and Condition (*p* < 0.001) for MT and a Group × Condition interaction (*p* < 0.01). Pair-wise comparisons of Group showed significant differences between no-history and concussion history for RT in condition VR (*p* < 0.05), VM (*p* < 0.05), VRM (*p* < 0.05) and HM (*p* < 0.01), and for MT in condition V (*p* < 0.05), VRM (*p* < 0.001), H (*p* < 0.05) and HM (*p* < 0.01). Both performance timing measures, RT and MT increased with increasing task difficulty, and were, across conditions, longer for concussion history participants compared to healthy controls with no-history of concussion (cf. Fig. 2a, b).

### Performance execution

Repeated-measures mixed ANOVA revealed significant effects of PL for Condition (*p* < 0.01), both not for Group and Group × Condition (both *p* > 0.05). Repeated-measures mixed ANOVA for CE (i.e. movement accuracy) revealed a main effect of Condition (*p* < 0.001), but no effects of Group and Group × Condition (both *p* > 0.05). Repeated-measures mixed ANOVA for VE (i.e. movement precision) revealed a main effect of Condition (*p* < 0.001), and an interaction between Group × Condition (*p* < 0.01), but no significant effect of Group (*p* > 0.05). Pair-wise comparisons showed no significant Group differences of PL for all variables (all *p* < 0.05), but significant differences between no-history and concussion history group for VE in condition V (*p* < 0.01), VR (*p* < 0.01), H (*p* < 0.001) and HR (*p* < 0.05). Increasing the task complexity lengthened the pathlength, and had a detrimental effect on endpoint accuracy and precision. Endpoint accuracy (CE) and endpoint precision (VE) decreased both with task difficulty for no-history and concussion history participants, however endpoint precision (VE) was lower for the participants with concussion history (cf. Fig. 2c).

### Direction reversals, and Task Completion Error Counts

Repeated-measures mixed ANOVA for direction reversals revealed no significant effects of Condition, Group or Group × Condition (all p > 0.05). We also did not find any significant group differences in the number of remaining error types (all p > 0.05).

### Performance as a Function of Level of Dissociation

Statistical outcomes of the repeated mixed measures ANOVA for the level of dissociation are summarized in Table 4, and descriptive statistics for all dependent variables of Group and Level are summarized in Table 5. We observed a main effect of Group (No-history, Concussion history) for RT (*p* < 0.05) and CE (*p* < 0.05), a main effect of Level (1 level, 2 levels) for MT (*p* < 0.001), and notably a significant Group × Level interaction for MT (*p* < 0.001) and VE (*p* < 0.05). Pair-wise comparisons of Group showed significant differences between no-history and concussion history participants of MT for 2 levels (*p* < 0.01), of CE for 1 level and 2 levels (both *p* < 0.05), and of VE for 1 level (*p* < 0.05).

**Table 4:** Statistical outcome of repeated-measures mixed ANOVA for Level (1 level, 2 levels) and Group (Concussion history, No-history)

Parameter	Group	Level	Group × Level
RT	F (1,33) = 4.55^*^	F (1,33) = 2.05^n.s.^	F (1,33) = 0.10^n.s.^
MT	F (1,33) = 3.77^n.s.^	F (1,33) = 25.05^***^	F (1,33) = 14.84^***^
PL	F (1,33) = 1.32^n.s.^	F (1,33) = 0.00^n.s.^	F (1,33) = 0.09^n.s.^
CE	F (1,33) = 6.509^*^	F (1,33) = 3.38^n.s.^	F (1,33) = 0.40^n.s.^
VE	F (1,33) = 2.76^n.s.^	F (1,33) = 2.85^n.s.^	F (1,33) = 5.75^*^
DR	F (1,33) = 0.23^n.s.^	F (1,33) = 0.00^n.s.^	F (1,33) = 0.49^n.s.^
Parameter	Level	Condition effect	
MT	2 levels	F (1,33) = 8.31^**^	
CE	1 level	F (1,33) = 5.54^*^	
CE	2 levels	F (1,33) = 5.45^*^	
VE	2 levels	F (1,33) = 6.53^*^	

Level represent dependent variable values averaged across one (VM, H, HM) and two levels (VR, VRM, HR) of dissociation between vision and action, distracted from V

Significant outcomes of pair-wise comparisons for Group (Concussion history, No-history) of all variables and both levels (1 level, 2 levels)

*RT* reaction time, *MT* movement time, *CE* endpoint accuracy, *VE* endpoint precision, *DR* direction reversal errors, *PL* pathlength, *V* vertical, *VR* vertical rotated, *VM* vertical memory, *VRM* vertical rotated memory, *H* horizontal, *HR* horizontal rotated, *HM* horizontal memory, *n.s*. non significant. Asterisks represent * < 0.05, ** < 0.01, *** < 0.001

**Table 5:** Descriptive statistics of the ANOVA for 1 level and 2 levels of disassociation for both groups (Concussion history, No-history)

Parameter	Level	Concussed	Non-concussed
		[ms]	[ms]
RT	1 level	59.42 ± 55.12	25.03 ± 50.98
	2 levels	49.43 ± 58.23	9.29 ± 66.83
MT	1 level	123.41 ± 79.55	111.67 ± 130.73
	2 levels	275.96 ± 152.01	131.53 ± 143.85
		[mm]	[mm]
PL	1 level	−1.26 ± 4.91	0.73 ± 3.48
	2 levels	−1.06 ± 6.67	0.54 ± 4.43
CE	1 level	4.20 ± 2.59	1.99 ± 2.97
	2 levels	5.29 ± 3.49	2.52 ± 3.53
VE	1 level	−0.13 ± 4.02	0.67 ± 1.61
	2 levels	−0.34 ± 3.35	1.92 ± 1.47
		[count]	[count]
DR	1 level	0.00 ± 0.30	0.12 ± 0.26
	2 levels	0.07 ± 0.72	0.06 ± 0.13

*RT* reaction time, *MT* movement time, *CE* endpoint accuracy, *VE* endpoint precision, *DR* direction reversal errors, *PL* pathlength, 1 *level* mean across conditions requiring 1 level of disassociation between vision and action, 2 *levels* mean across conditions requiring 2 levels of disassociation between vision and action

**Fig. 3:**
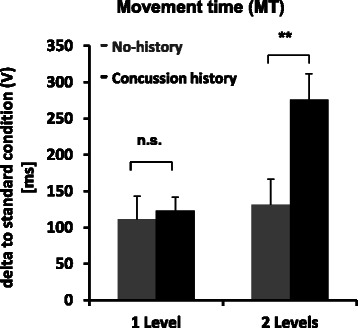
Mean movement time values as a function of dissociation from direct interaction. Movement time (MT), presented as function of 1 level and 2 levels of dissociation between vision and action subtracted from the standard mapping condition (V), for both groups (Concussion history, No-history). Note the prolonged movement time for participants with concussion history compared to no-history participants for two levels of dissociation, but not in one level. Abbreviations: n.s. = non-significant, asterisks represent **p < 0.01. Error bars represent standard error of the mean

Most importantly, the change between non-standard-mapping and standard mapping condition for MT increased dramatically in the concussion history group in going from 1 level of dissociation to 2 levels of dissociation, while there was no significant MT increase in no-history control participants’ MT (cf. Fig. 3 and Table. 5). Across groups, the change between non-standard-mapping and standard mapping condition for MT was significant longer in the 2 level (203.743 ± 25.05 ms) than in the 1 level (117.54 ± 18.17 ms) condition. Across levels, the change between non-standard-mapping and standard mapping condition for RT was significantly longer in the concussion history (54.42 ± 12.17 ms) compared to no-history of concussion group (17.17 ± 12.52 ms), and movement accuracy (CE) was significantly less in the concussion history group (4.75 ± 0.68 mm) compared to no-history controls (2.25 ± 0.70 mm). Interestingly, movement precision (VE) showed the opposite effect, increasing from a very small amount (precise) for no-history control participants in the 1 level condition to a much greater amount (imprecise) in the 2 level condition (*p* < 0.05), an increase not seen for concussion history participants, whose values were in the middle range relative to no-history control performance (cf. Tab. 5).

### Discriminant analysis

**Fig. 4:**
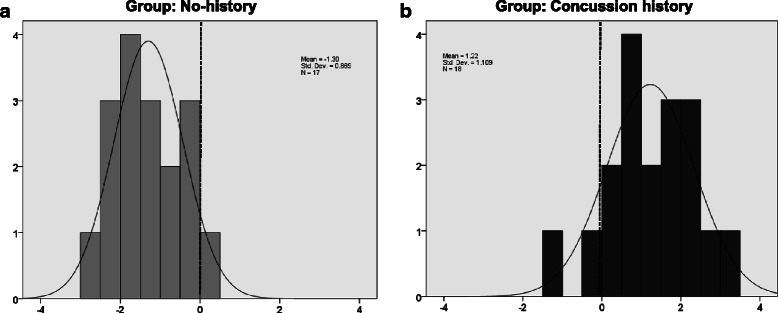
Stepwise discriminant analysis results. Normal distribution of discriminant scores for No-history (**a**) and Concussion history (**b**) participants. Scores are a weighted contribution of dependent variables chosen by the analysis to maximize differentiation between groups. Note the good separation between concussion history and no-history of concussion data points

The discriminant analysis performed for the concussion history group demonstrated good separation from the no-history control group. Based on the outcome of the dependent variable analyses, the predictors supplied to the discriminant function classifying concussion history *versus* no-history control participants were VE and MT in the H condition, and MT in the VRM condition. Using these variables, the outcome of the discriminant analyses showed that our assessment tool was able to discriminate athletes with a history of concussion from athletes with no-history of concussion with an accuracy of 94 % (for details please see Table. 6 and Fig. 4).

**Table 6:** Classification results ^a,c^ of stepwise discriminant analyses

		Group	Predicted Group Membership		Total
			Conc.	Control	
Original	Count	Conc.-H.	16	1	17
		No-H.	2	16	18
	%	Conc.-H.	94.1	5.9	100
		No-H.	11.1	88.9	100
Classification^b^	Count	Conc.-H.	15	2	17
		No-H.	2	16	18
	%	Conc.-H.	88.2	11.8	100
		No-H.	11.1	88.9	100

^a^91.4 % of original grouped cases correctly classified

^b^Cross validation is done only for those cases in the analysis. In cross validation, each case is classified by the functions derived from all cases other than that case

^c^88.6 % of cross-validated grouped cases correctly classified

*Conc*.-*H*. concussion history, *No*-*H*. no-history of concussion

## DISCUSSION

The main aim of this study was to determine whether participants with a history of concussion had deficits in a cognitive-motor integration task when compared to healthy controls without a history of concussion. A second aim of this study was to assess the effectiveness of our computer-based task to detect cognitive-motor integration deficits in participants with concussion history, which has proven effective at quantifying subtle cognitive-motor integration changes in those at risk of, or in the early stages of dementia [11–13, 16]. We observed differences in performance on complex visuomotor tasks between varsity-level athletes with a history of concussion and healthy age-matched adults with no-history of concussion. Specifically, participants with a concussion history had difficulty executing visually-guided movements when there was a single level of dissociation between the guiding visual information and the required motor action. In support of our hypothesis, participants with a concussion history also displayed both impaired movement planning and execution when the brain had to control a movement with the highest level of dissociation between vision and action. Notably, our assessment tool was able to discriminate between athletes with a history of concussion – most of whom are asymptomatic by current measures–and athletes with no-history of concussion with 94 % accuracy.

Our present findings complement those of our previous studies on adults at-risk (through an MCI diagnosis or family history) for the development of Alzheimer’s disease [12, 13]. While similar behavioural deficits between these otherwise very different groups do not mean the mechanism underlying motor impairment is the same, it does suggest that young athletes with a history of concussion are neurologically fragile, even when they are asymptomatic and in the late period of the return to play/work schedule. A task which ‘pushes the system’ appears to bring out behavioural deficits across a range of mild brain dysfunction. We propose that the present results reflect a problem in the *communication* between brain regions responsible for planning and executing skilled movement when there is an element of cognition involved. This proposal is based on recent work on brain networks for cognitive-motor integration in both human-and non-human primates [14, 18–24]. In particular, our behavioural results may arise from impairments in parietal-frontal networks as well as communication between cortical and subcortical movement control regions due to cellular damages following concussion. Cellular changes are well known following concussion, with an initial reduction in cerebral blood flow and cerebral glucose utilization followed by membrane depolarization and excess excitatory neurotransmitter release [25–28]. Such changes could lead to problems in ATP metabolism [28] and in long-term potentiation (LTP) of the brain [29–31]. While the long-term effects of subtle hypoxia on brain tissue are not fully understood, it is becoming increasingly clear that oxidative stress may trigger neuropathological processes both local and distal to the site of mild brain contusion, effects which may only manifest themselves months to years later [31]. Indeed, recent studies of brain metabolism in adults with concussion history have found concurrent motor skill deficits and primary motor cortex metabolism abnormalities related to deficits in motor learning [32]. Lingering abnormalities in not only individual brain areas but the pathways between them may thus underly problems in more complex behaviour manifested following concussion.

In support of our proposal, imaging and brain function studies have found alterations in both function and anatomy in particular brain regions following concussion. Studies have found increased and more widespread activation of pre-frontal cortex (PFC), dorsolateral prefrontal cortex, and cerebellum for a variety of tasks in concussed *versus* non-concussed populations [3–5]. These authors suggested that the additional and more elaborate network activations may reflect compensatory mechanisms to accommodate functional and/or structural deficits in the brain’s default networks as a result of concussion. In terms of networks that may relate specifically to cognitive-motor integration, imaging studies have found increased activation in parietal, frontal, as well as cerebellar regions in concussed individuals compared to pre-injury fMRI data, although no cognitive performance deficits were noted [33]. Further, recent anatomical studies have found altered white matter integrity in concussed adolescents, younger adults with concussion history, and older adults with concussion history that involved those pathways connecting frontal and parietal regions [34–37]. These data complement our group’s findings on the crucial role played by such fronto-parietal networks in integrating thought and action [10, 14, 19, 20, 38].

A notable aspect to the group of athletes examined in this study is that the majority of our athletes with a concussion history were classified as “asymptomatic” by current testing standards (computerized cognitive testing alone, balance/coordination testing alone), which suggests that the current testing standards are examining individual brain regions. When one is forced to integrate one's behavior across the traditionally segregated domains of cognition and action, something which athletes and skilled workers are often called upon to do in the course of their actual activities, the integrity of brain connections between regions is likely crucial for successful performance. We suggest that multi-domain tasks such as the one used here are more effective at assessing function prior to a return to activity, in that they allow one to test communication between brain regions needed for the control of complex skilled action. Further studies are needed to better understand the detailed neural mechanisms that are involved in cognitive-motor integration following concussion. These further studies should taking into account number of concussions and aspects of one’s medical history, specifics of the concussive event itself, as well as severity and time since last concussion.

## Conclusions

The present data suggest that our cognitive-motor integration task is able to sensitively detect concussion-related functional performance changes, important for the safe return to play/work. Additionally, our assessment tool was able to discriminate between athletes with concussion history–most of whom are asymptomatic by current measures–and athletes with no-history of concussion with 94 % accuracy. This type of evaluation in domains crucial for safe performance (e.g. sports, military, industry, rehabilitation settings) represents a clinically efficient approach to concussion recovery assessment. Based on our “level of disassociation” analysis, it is feasible to reduce the experiment conditions used here (i.e., one standard-mapping condition and one non-standard mapping condition requiring two levels of disassociation), to allow a simple, quick assessment tool for the both clinical and field settings.
